# Diagnostic accuracy of combined thoracic and cardiac sonography for the diagnosis of pulmonary embolism: A systematic review and meta-analysis

**DOI:** 10.1371/journal.pone.0235940

**Published:** 2020-09-28

**Authors:** Jacqueline Kagima, Marie Stolbrink, Sheila Masheti, Collins Mbaiyani, Aziz Munubi, Elizabeth Joekes, Kevin Mortimer, Jamie Rylance, Ben Morton

**Affiliations:** 1 Department of Clinical Sciences, Liverpool School of Tropical Medicine, Liverpool, United Kingdom; 2 Department of Internal Medicine, Kenyatta National Hospital, Nairobi, Kenya; 3 Aintree University Hospital NHS Foundation Trust, Liverpool, United Kingdom; 4 Kenya Medical Research Institute, Centre for Respiratory Disease Research, Nairobi, Kenya; 5 Department of Health Management and Informatics, Kenyatta University, Nairobi, Kenya; 6 Department of Radiology, Royal Liverpool University Hospitals NHS Trust, Liverpool, United Kingdom; 7 Lung Health Group, Malawi-Liverpool-Wellcome Programme (MLW), Blantyre, Malawi; BronxCare Health System, Affiliated with Icahn School of Medicine at Mount Sinai, UNITED STATES

## Abstract

**Objectives:**

Computed tomography pulmonary angiography (CTPA) is the diagnostic standard for pulmonary embolism (PE), but is unavailable in many low resource settings. We evaluated the evidence for point of care ultrasound as an alternative diagnostic.

**Methods:**

Using a PROSPERO-registered, protocol-driven strategy (https://www.crd.york.ac.uk/PROSPERO, ID = CRD42018099925), we searched MEDLINE, EMBASE, and CINHAL for observational and clinical trials of cardiopulmonary ultrasound (CPUS) for PE. We included English-language studies of adult patients with acute breathlessness, reported according to PRISMA guidelines published in the last two decades (January 2000 to February 2020). The primary outcome was diagnostic accuracy of CPUS compared to reference standard CTPA for detection of PE in acutely breathless adults.

**Results:**

We identified 260 unique publications of which twelve met all inclusion criteria. Of these, seven studies (N = 3872) were suitable for inclusion in our meta-analysis for diagnostic accuracy (two using CTPA and five using clinically derived diagnosis criterion). Meta-analysis of data demonstrated that using cardiopulmonary ultrasound (CPUS) was 91% sensitive and 81% specific for pulmonary embolism diagnosis compared to diagnostic standard CTPA. When compared to clinically derived diagnosis criterion, CPUS was 52% sensitive and 92% specific for PE diagnosis. We observed substantial heterogeneity across studies meeting inclusion criteria (I^2^ = 73.5%).

**Conclusions:**

Cardiopulmonary ultrasound may be useful in areas where CTPA is unavailable or unsuitable. Interpretation is limited by study heterogeneity. Further methodologically rigorous studies comparing CPUS and CTPA are important to inform clinical practice.

## Introduction

Pulmonary embolism (PE) is a significant public health problem with an estimated 10 million cases per year worldwide [1]. Despite advances in diagnosis and therapy, PE is frequently undiagnosed and untreated; especially in low resource settings [2]. Computed tomography pulmonary angiography (CTPA) is currently considered the diagnostic standard for the diagnosis of PE [3, 4]. However, CTPA is not always available, affordable or feasible, particularly in LMIC settings. A multiorgan ultrasonographic approach has been suggested as a pragmatic alternative diagnostic approach for PE but there is currently a lack of evidence on diagnostic accuracy to inform adoption [5].

A combination of lung ultrasound (LUS) and cardiac echocardiography has been advocated in patients with suspected pulmonary embolism [4, 6]. Ultrasound techniques may be particularly relevant in low-resource settings as this modality is more accessible and less expensive than CTPA. Ultrasound is portable, noninvasive, repeatable, and has no risk of ionizing radiation [7]. Additionally, the use of ultrasound has been shown to promote patient safety [8]. However, use of ultrasonography requires appropriate training and quality assurance. Misinterpretation leads to diagnostic inaccuracy, inappropriate treatment and potential for harm from e.g. unnecessary or delayed anticoagulation. Important barriers to the widespread uptake of ultrasonography include lack of training and a limited evidence base [9].

Our aim was to determine the diagnostic accuracy of cardiopulmonary point of care ultrasound in the diagnosis of pulmonary embolism in acutely breathless adults. To do this, we systematically reviewed and synthesized the published literature. There are meta-analyses in the existing literature establishing the accuracy of a single-organ scan (LUS or cardiac echocardiography applied alone), and in combination with other add-on laboratory and imaging tests for PE diagnosis. To date, no study has systematically reviewed the literature for a multi-organ point of care ultrasound that combines LUS and cardiac echocardiography for PE diagnosis in dyspneic adult patients.

## Materials and methods

We followed the preferred reporting items for systematic reviews and meta-analyses (PRISMA) statement [10]. The checklist is provided in the data in S1 Appendix. The review protocol and search strategy were registered with PROSPERO (http://www.crd.york.ac.uk/PROSPERO, ID: CRD42018099925).

### Data sources and search strategy

The initial systematic search of MEDLINE, EMBASE, and CINAHL databases for English language papers was conducted on 6^th^ June 2017 and updated on 28^th^ February 2020. We included studies published from 1^st^ January 2000 until 28^th^ February 2020. The bibliography of included studies was searched for any additional relevant titles. Combinations of subject headings, keywords and synonyms used included: breathless* OR dyspn* OR “shortness of breath”; “pulmonary embol*” OR “pulmonary thromboembol*”; sonogr* OR ultraso* OR “point‐of‐care ultraso*” OR and “bedside ultraso*” (further details provided in the data in S2 File).

### Study selection and data extraction

**Fig 1:**
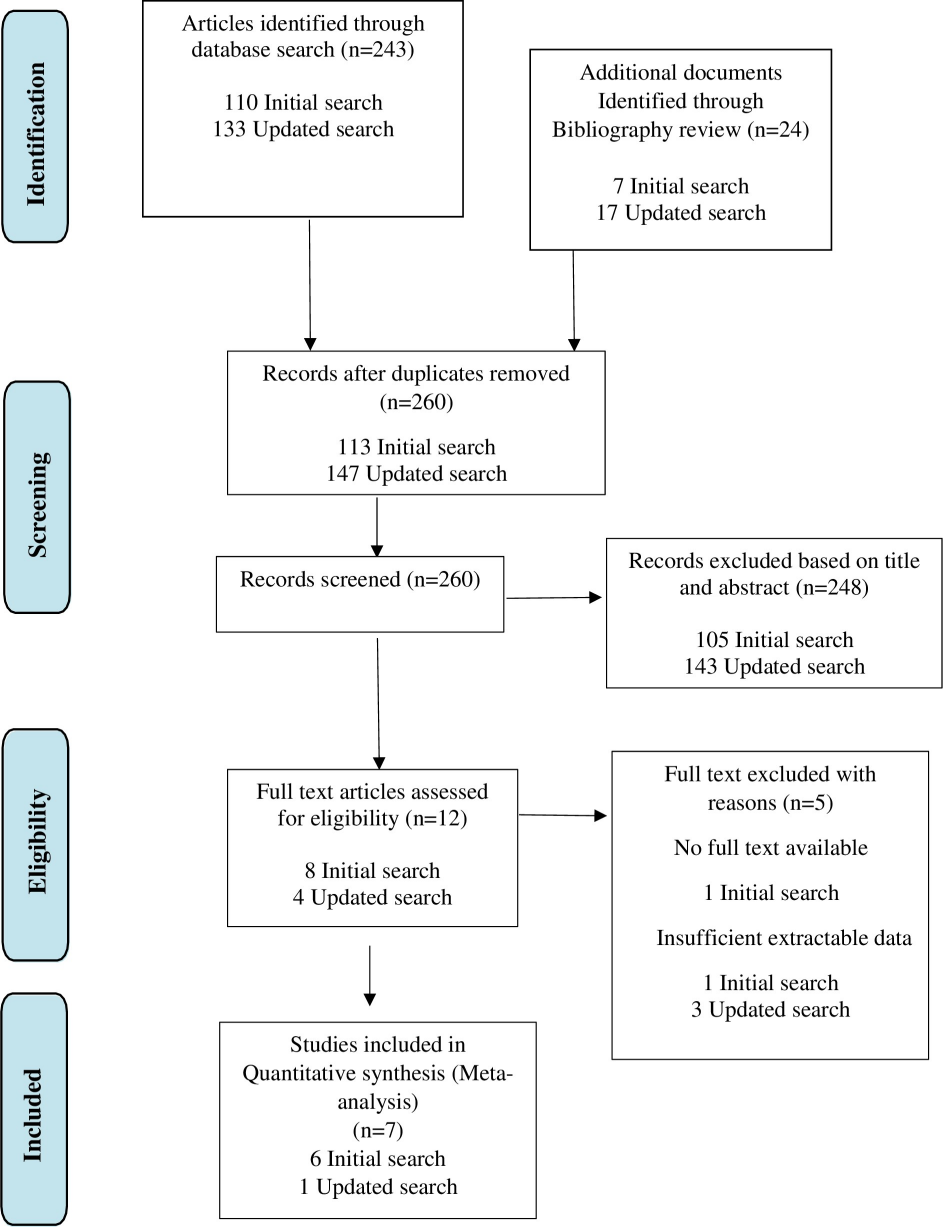
PRISMA flow diagram showing the study selection process.

We included prospective and retrospective observational studies and clinical trials that recruited acutely breathless adults (≥ 18 years). Non-English papers, unpublished research, conference communications, pediatric studies and studies on animals were excluded. We considered CTPA as the diagnostic standard for PE, but we also included studies that incorporated clinically derived diagnosis as a reference standard. Data was extracted on the study setting, sampling methods, characteristics of the study design, reference standard used in the diagnosis of PE and additional tests done, type of ultrasound machine used, and the organs scanned and sonographers’ qualifications, experience and blinding. The diagnostic accuracy measure was our primary outcome measure. Two reviewers (JK and BM) screened titles and abstracts and made study selection decisions independently. Papers that at least one reviewer identified for inclusion were reviewed in full (Fig 1). Data retrieved from these studies by both researchers were compared. Where these were not provided in the original publication, we sent requests for raw data to the authors of studies. Disagreements were resolved by consensus between reviewers (JK and BM) or final arbitration by JR.

### Quality assessment

Methodological quality was assessed through the QUADAS-2 (Quality Assessment of Diagnostic Accuracy Studies) criterion [11], which categorized the risk of bias involving methodology, reporting and validity as low, unclear or high.

### Data analysis

Data were extracted as two‐by‐two tables (true positives, false positives, true negatives, and false negatives) and analysis conducted using Review Manager 5 (revman) version 5.3 [12] and R-CRAN project version 3.6.3. [13] using the R package ‘meta’ [14]. Since patients are likely to have more than one abnormal finding per ultrasound, we used the individual patient as the unit of analysis (abnormal finding versus none) and not the individual ultrasound findings.

### Assessment of heterogeneity

Study heterogeneity was evaluated with Cochrane’s Q and quantified with the inconsistency (I^2^) test, using a random effects model. We also assessed heterogeneity by visually inspecting the forest plots to determine closeness of point estimates with each other and overlap of confidence intervals (CIs).

### Publication bias

To assess for publication bias, we used funnel plot [15] for a random-effects model and also performed a regression test with a meta-regression model (Egger's regression test) [16] using R software to assess for plot asymmetry.

## Results

### Study selection

We identified 260 unique titles of which 248 were excluded during the title and abstract screening process. Following full text review of the remaining 12 articles, five did not meet inclusion criteria for various reasons (see Fig 1 below). Seven articles [17–23] could be included in the meta-analysis, incorporating 3872 patients. Two papers used CTPA as the reference diagnostic standard [19, 20], and five papers used clinically derived diagnosis as the reference standard [17, 18, 21–23] Clinically derived diagnosis adjudicators were blinded from the CPUS results in each of these studies. One study of acutely breathless patients [20] identified PE by exclusion following ultrasound examination for other candidate diagnoses.

### Study characteristics

The main characteristics of eligible studies, which included between 96 and 2683 patients (pooled total 3872) are summarized in Table 1. The majority of the studies were published between 2013 and 2017, and with the exception of one [19] were carried out in single centers [17, 18, 20–23] Six of the included studies were done in emergency departments (ED) and one was done in an intensive care unit (ICU) [21] all in high income country settings (Table 1). All included studies used at least one of the following alternate tests in their clinical diagnosis: blood gases, D-Dimers, chest radiograph (CXR), troponins, brain natriuretic peptide (BNP) or computed tomography (CT) chest. In all studies the sonographers were blinded to the laboratory results, although these were available to inform clinically derived diagnosis.

**Table 1:** Baseline characteristics of included studies.

Author	Year	Country	Study setting	Sampling	Design	Reference standard	Sample size (n)	Leg US	Ultrasound machine	Sonographer qualifications
Laursen [17]	2013	Denmark	Single ED	Convenient	Prospective	Clinically derived diagnosis	134	Yes	Vivid S6	1 Dedicated physician; Done
									GE	
					Cohort					
										>100 FATE and LCUS scans;
										>300 FLUS scans
Laursen [18]	2014	Denmark	Single ED	Consecutive	RCT	Clinically derived diagnosis	158	Yes	Logiq S8	1 Dedicated physician; Done >200 CPUS scans.
									GE	
Bataille [21]	2014	France	Single ICU	Consecutive	Prospective	Clinically derived diagnosis	136	No	Sonos 5500	Not reported
					Cohort				Philips HP	
Nazerian [19]	2014	Italy	Multiple ED	Consecutive	Prospective	CTPA	357	Yes	3 MyLab30 Gold, 1 MyLab40 (Esaote SpA); 1 Logiq3, GE; 1 HD7 (Philips)	9 EPs with at least 5 years’ experience and 4 residents (2 IM and 2EM.)
					Cohort					
Koenig [20]	2014	USA	Single ED	Convenient	Prospective	CTPA	96	Yes	M-turbo	3 pulmonary critical care faculty and 3 third-year fellows.
					Cohort				Sonosite	
Zanobetti [22]	2017	Italy	Single ED	Consecutive	Prospective	Clinically derived diagnosis	2683	No	MyLab 30 Gold Esaote SpA	10 EPs having attended 80 hours course and done 150 CPUS with 2 years’ experience.
					Cohort					
Jung [23]	2017	Korea	Single ED	Consecutive	Prospective	Clinically derived diagnosis	308	No	ACUSON X500 Siemens	Board certified EPs with > 5 years’ experience in CPUS and 2 senior EM residents.
					Cohort					

CPUS, cardiopulmonary ultrasound; CTPA, computed tomography pulmonary angiography; ED, emergency department; EM, emergency medicine; EP, emergency physician; FATE, focus-assessed transthoracic echocardiography; FLUS, focused lung ultrasound; IM, internal medicine; ICU, intensive care unit; LCUS, leg compression ultrasound; RCT, randomized controlled study; US, ultrasound.

Five studies [18, 19, 21–23] enrolled a consecutive sample, and two studies [17, 20] enrolled a convenience sample (Table 1). Only one study included two study arms (randomized control design) [18], of which only the intervention arm included CPUS; we excluded those patients allocated to the control arm. In six studies [17–20, 22, 23], the sonographer was an ED physician, pulmonary or critical care faculty, fellow or resident. The minimum ultrasound experience level varied markedly between studies, ranging from at least two years’ experience to more than 10 years’ experience. In three studies, the qualification of the sonographer was reported in terms of the minimum number of ultrasound procedures performed, which ranged from 100 to more than 400 [17, 18, 22]. Sonographer experience was not described in one study [21].

Five studies [17, 18, 21–23] used clinically derived diagnosis established by one or more independent adjudicators as the reference standard. In four of these studies [17, 18, 21, 23], the clinical experts who adjudicated the final diagnosis were blinded to the ultrasound findings; however, in one study [22], these clinicians were not blinded to the ultrasound results. Two studies considered as the reference standard exclusively the CTPA scan [19, 20].

### Assessment of methodological quality

#### Risk of bias and applicability concerns within studies

**Fig 2:**
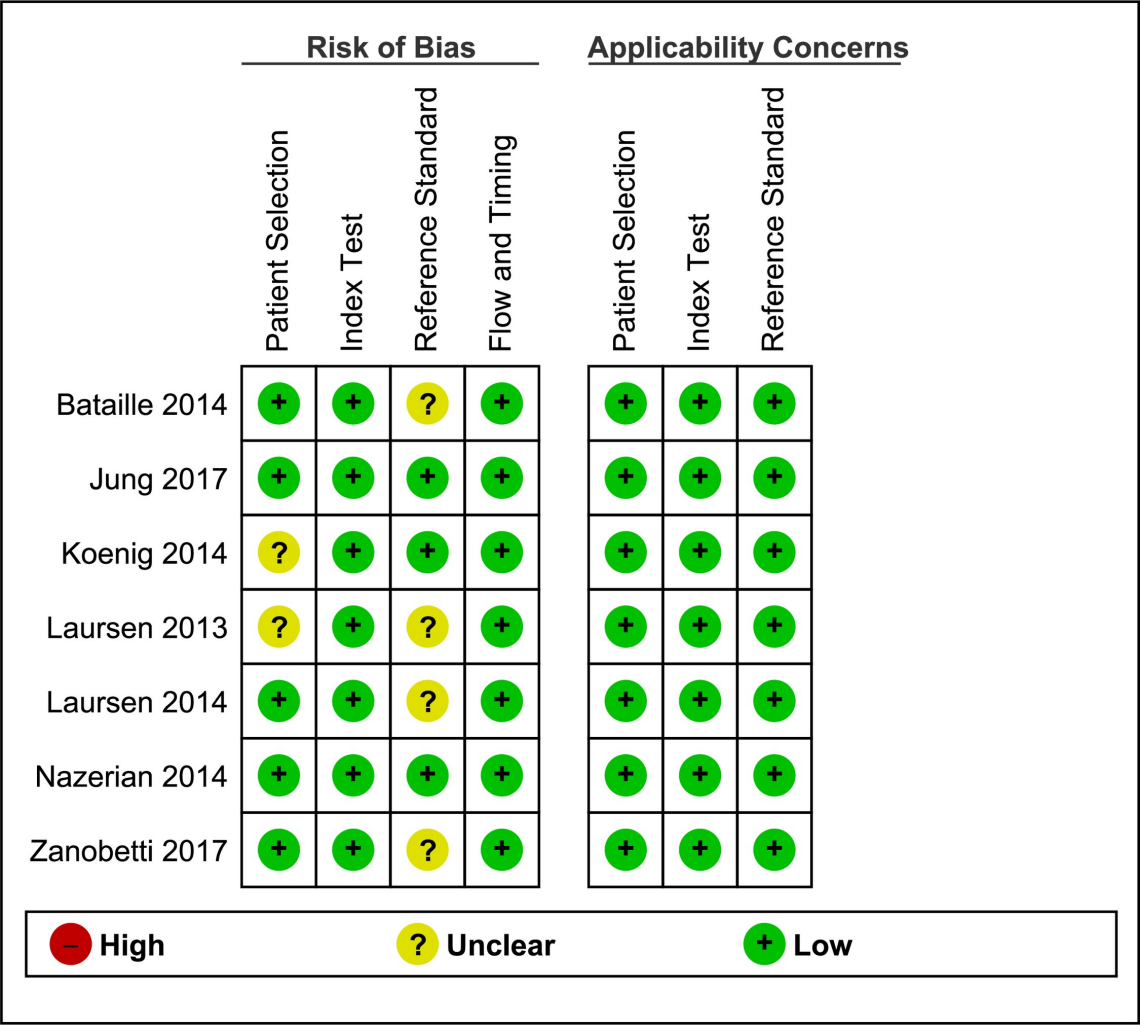
Risk of bias and applicability concerns summary. Review of authors' judgments about each domain for each included study. Majority of the studies had a low risk of bias.

**Fig 3:**
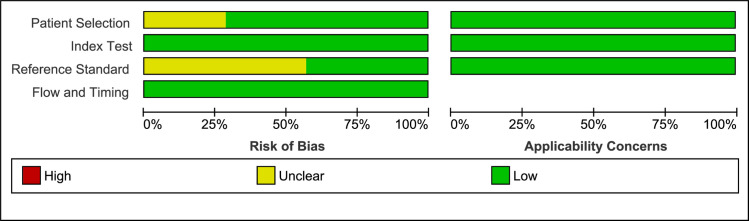
Risk of bias and applicability concerns graph. Review of authors' judgments about each domain presented as percentages across included studies Majority of the studies had a low risk of bias.

The quality of all studies was generally high, had low risk of bias, and satisfied the majority of the risk of bias and applicability criteria. Quality assessment of individual studies is summarized in Figs 2 and 3 below.

### Sensitivity and specificity reported by individual studies

**Fig 4:**
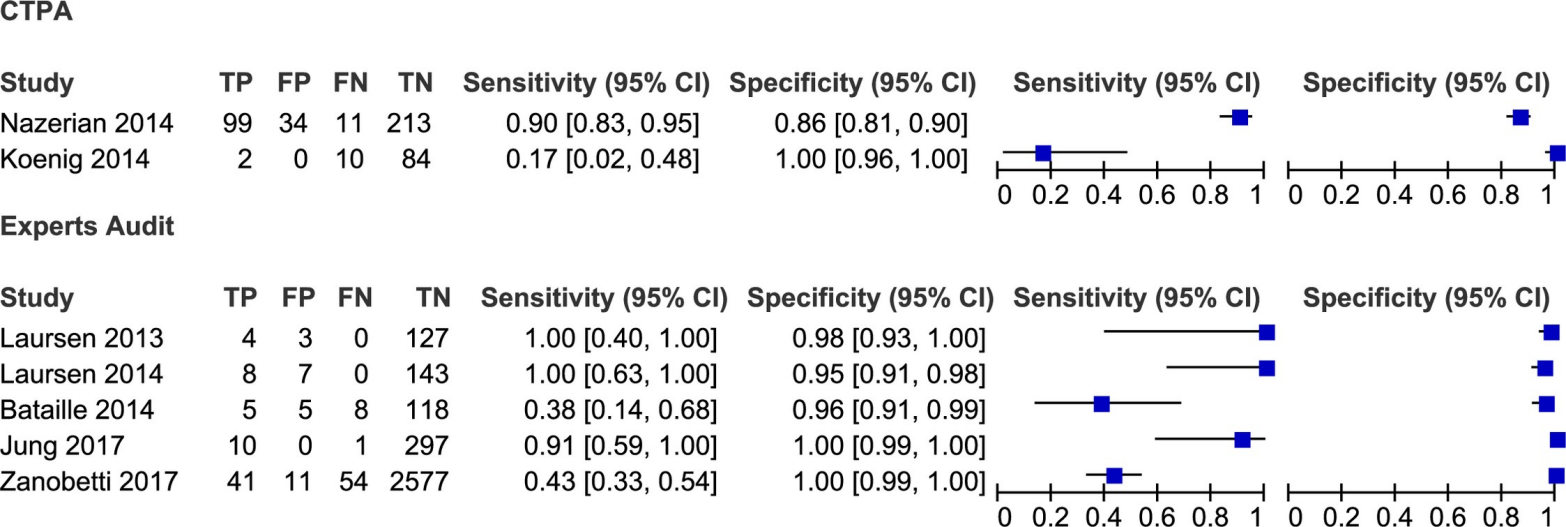
Forest plot of sensitivity and specificity of the individual studies. Forest plots presenting the estimates of sensitivity and specificity and 95% credibility intervals of each study across the two reference standards used (CTPA and clinically derived diagnosis). FN, false negative; FP, false positive; TN, true negative; TP, true positive.

The sensitivity for the studies that used a CTPA reference standard ranged from 17% (95% CI = 2%-48%) to 90% (95% CI = 83%-95%), with specificity from 86% (95% CI = 81%-90%) to 100% (95% CI = 96%-100%). For those studies that used clinically derived diagnosis as reference standard, sensitivity ranged from 38% (95% CI = 14%-68%) to 100% (95% CI = 40%-100%), with specificity from 95% (95% CI = 91%-98%) to 100% (95% CI = 99%-100%) (Fig 4 below).

### Synthesis of results

**Fig 5:**
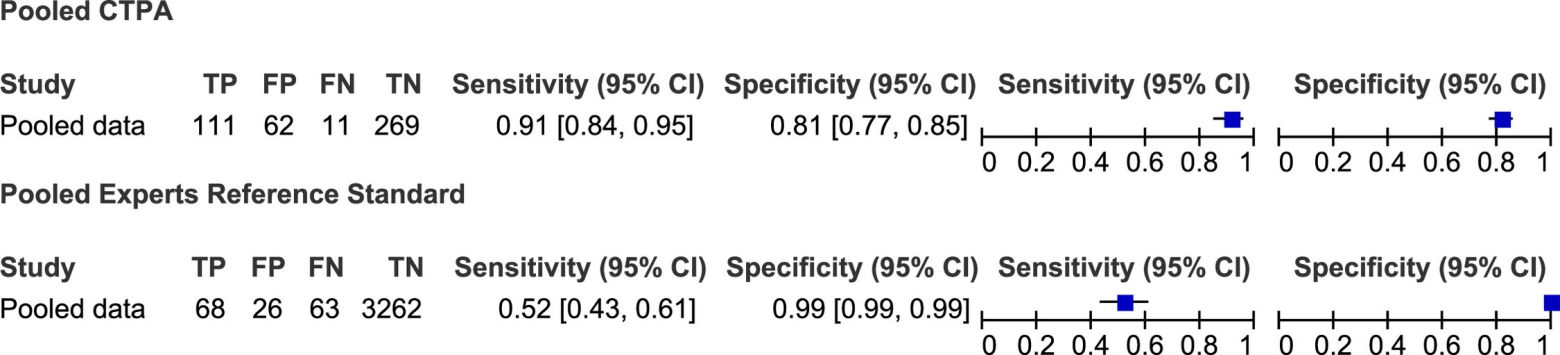
Forest plot of pooled estimates of sensitivity and specificity of the reference standard modalities. Forest plots presenting the pooled estimates of sensitivity and specificity and 95% credibility intervals of the two reference standards used (CTPA and clinically derived diagnosis). FN, false negative; FP, false positive; TN, true negative; TP, true positive.

**Fig 6:**
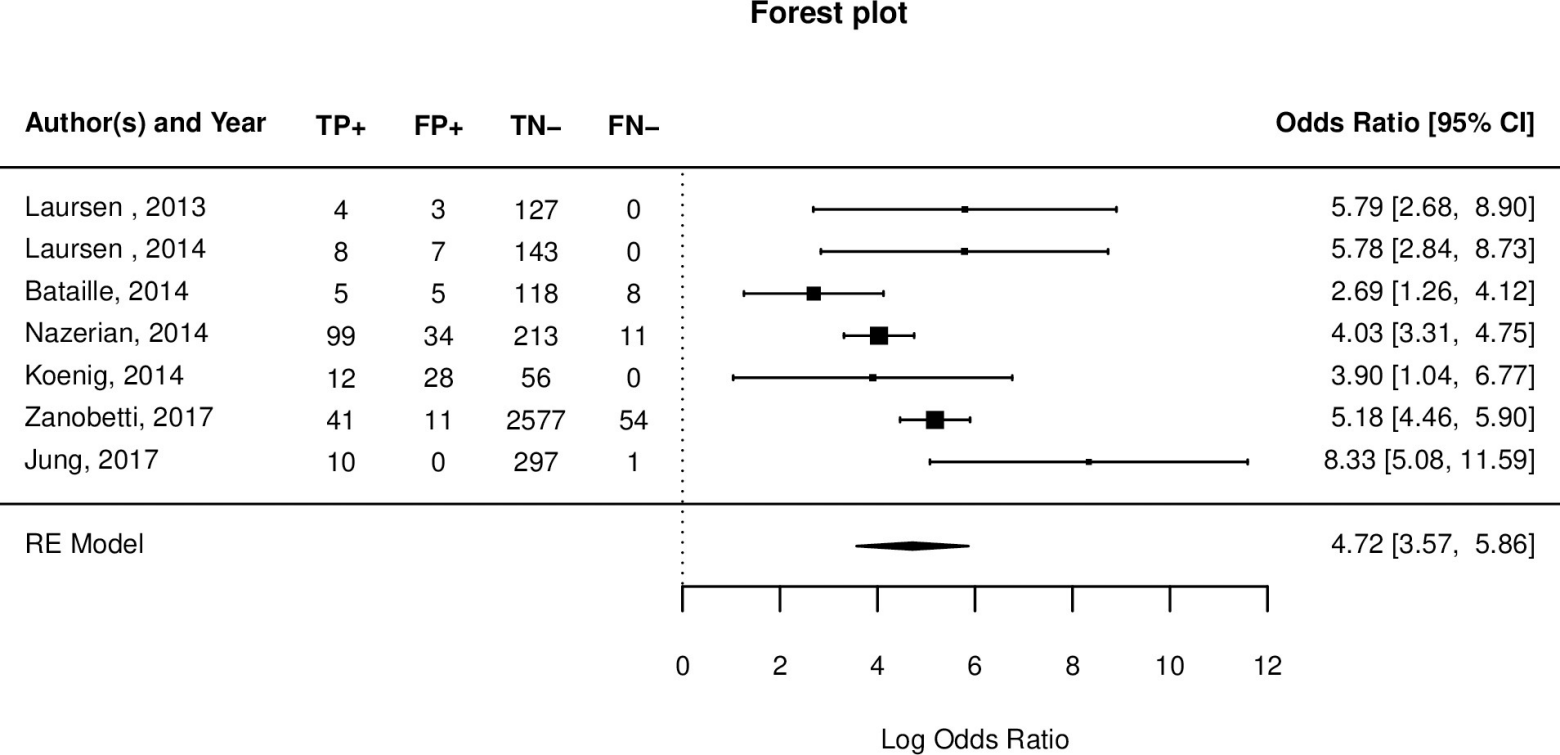
Forest plot of pooled diagnostic odds ratio of CPUS in diagnosing PE. Forest plots presenting the odds ratios and 95% credibility intervals of all the studies (FN, false negative; FP, false positive; TN, true negative; TP, true positive.

The pooled estimate of the sensitivity of CPUS for detection of PE based on CTPA as the reference standard was 91% (95% CI = 84%-95%) and specificity was 81% (95% CI = 77%-85%). The pooled sensitivity and specificity for detection of PE using clinically derived diagnosis as the reference standard was 52% (95% CI = 43%-61%) and 99% (95% CI = 99%-99%) respectively (Fig 5 below). The pooled diagnostic log odds ratio was 4.72 (95% CI:3.5 to 5.86) as shown on Fig 6 below.

### Heterogeneity assessment

**Fig 7:**
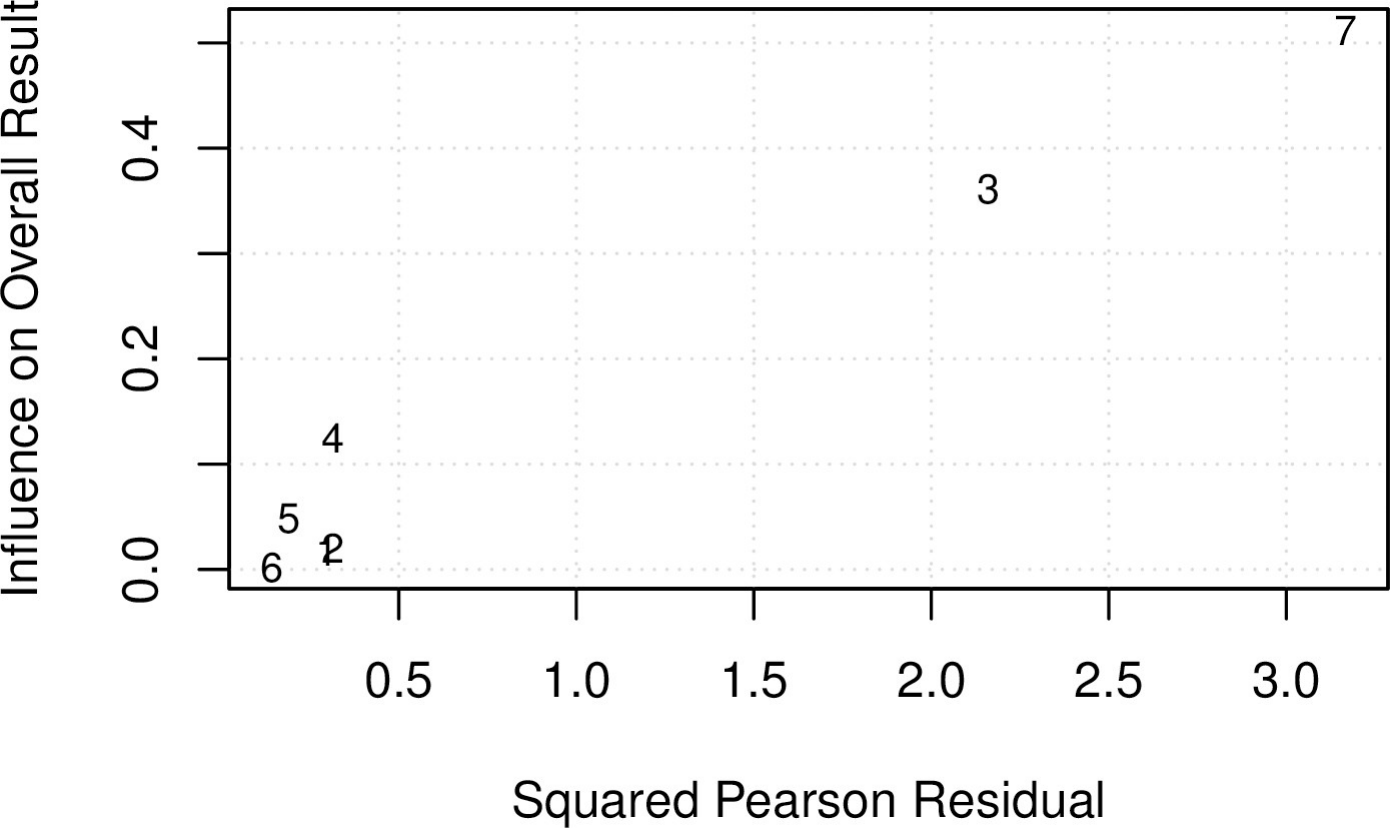
Baujat plot. 1-Laursen 2013, 2-Laursen 2014, 3- Bataille, 4- Nazerian, 5- Koenig, 6-Zanobetti, 7-Jung. Studies that fall to the top right quadrant of the Baujat plot contribute most to both the overall heterogeneity and overall results.

Significant heterogeneity was found across studies (Q (df = 6) = 18.0788, p-value = 0.006), of moderate magnitude (I^2^ = 73.5%). Total heterogeneity, as estimated by tau^2^, was 1.4 (95% CI:0.11 to 14.15). Studies by Bataille (performed in ICU) [21] and Jung (in ED) [23] contributed most to both overall heterogeneity and results as demonstrated by the Baujat plot [24] below (Fig 7). The former study did not indicate the qualifications of the sonographer.

### Publication bias

**Fig 8:**
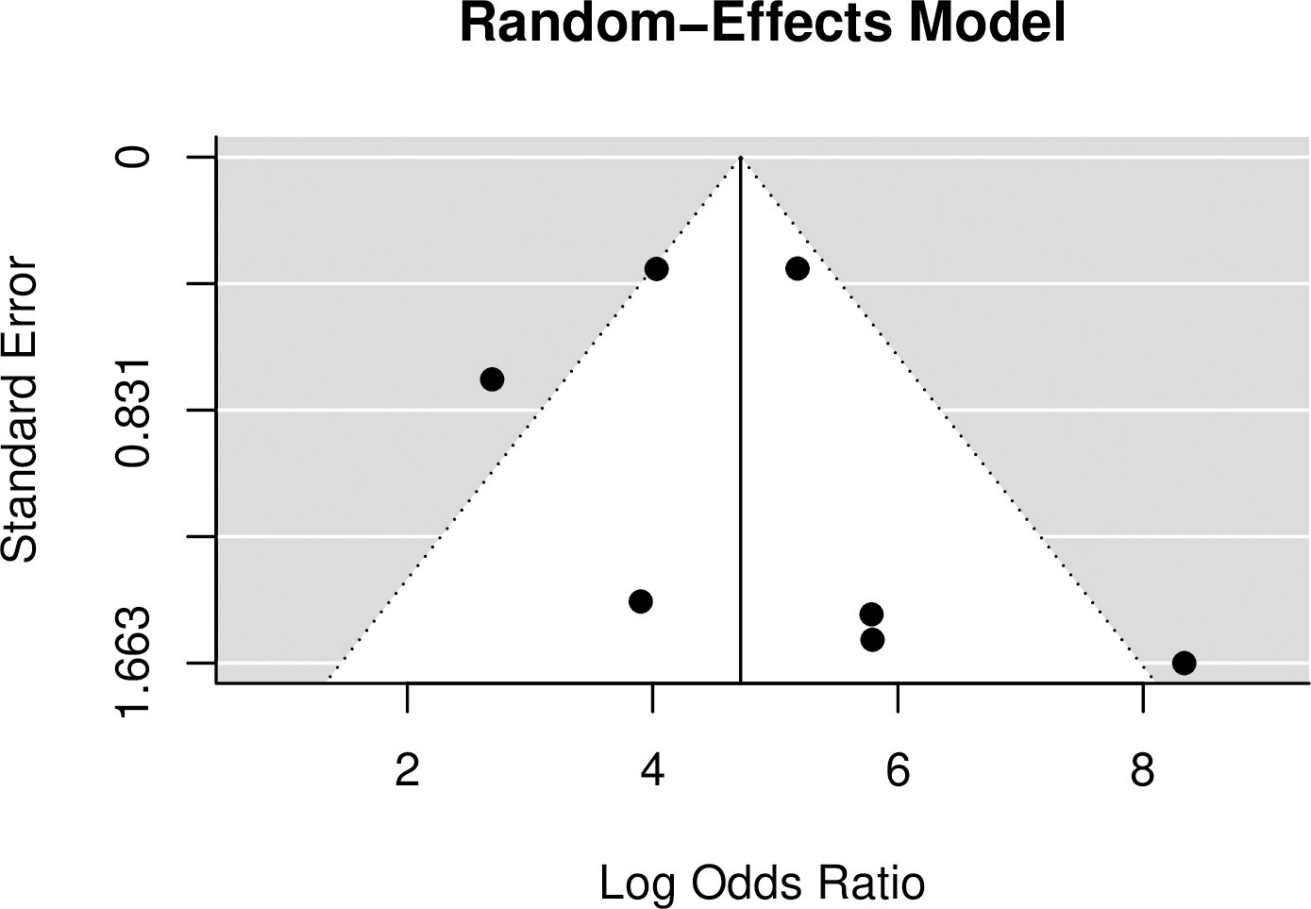
Funnel plot for the random effects model. Plot shows symmetry.

The funnel plot for a model without moderators (random-effects model), demonstrated symmetry, and no evidence of publication bias (Fig 8), and Egger's regression test [16] for plot asymmetry was consistent with this (p = 0.21).

## Discussion

The findings of this systematic review suggest that cardiopulmonary ultrasound could play an important role in PE diagnostics. In this meta-analysis we found that cardiopulmonary ultrasound (CPUS) is 91% sensitive and 81% specific for a diagnosis of PE when compared to the diagnostic standard CTPA. We identified a number of studies that compared ultrasound with clinically derived PE diagnosis; interpretation of such studies, where the clinical reference standard is known to be poorly sensitive and specific compared to CTPA, is challenging. Optimal PE diagnostics may not be feasible in specific settings such as when CTPA is unavailable or patient transfer is unsafe, and it appears that the CPUS may offer a viable alternative.

Previous metanalyses found that the sensitivities of single organ ultrasonographic test were lower than those of CPUS. Echocardiography used as a single ultrasonographic test in PE diagnostics in critically ill adults showed a 53% sensitivity and 83% specificity in a recent meta-analysis [25]. LUS used as a single ultrasonographic test to diagnose PE in adults admitted to EDs and medical wards showed an 87% sensitivity and 81.8% specificity [26]. These results confirm the limitation of a single-organ ultrasonography to rule out PE. The Bedside Lung Ultrasound in Emergency (BLUE) protocol is a protocol that allows diagnosis of PE based on a positive vein ultrasonography in combination with the exclusion by lung ultrasonography of other causes of respiratory failure, introduced by Lichtenstein and Mezière. The BLUE protocol showed a sensitivity of 81% and a specificity of 99% for PE diagnostics [27]. BLUE protocol has also been tested in combination with echocardiography in a small study performed in ED patients. None of the patients with an alternative ultrasonographic finding had PE on CTPA, thus showing an added value of CPUS in PE diagnostics [20]. These studies show a consistently higher specificity compared to the sensitivity and this could be due to the inability to rule out PE without the CTPA. Nevertheless, these findings suggested that the use of ultrasound has the potential to reduce the number of unnecessary CTPAs [6].

As with any ultrasonographic test, ultrasound is operator dependent. Specific training is required, and the experience of the sonographer is necessary to increase test accuracy. There are often incidental findings on CTPA which may or may not have clinical significance, but which increase anxiety, use of further diagnostic tests and consume clinical and patient time and resources [28]. The European Society of Cardiology (ESC) suggests that the best diagnostic strategy to confirm or exclude PE is to combine clinical assessment, plasma D-dimer measurement and computed tomographic pulmonary angiography (CTPA) [29]. As with all diagnostic tests, the accuracy of each diagnostic method is highly enhanced when used in conjunction with others. Considering this, a multi-organ ultrasound approach ‘triple scan' has been proposed which includes the use of cardiac, lung, and leg compression ultrasound scan. A proposed algorithm for the diagnosis of PE when other imaging diagnostics are not available is included in the data in S2 Fig. However, there is no robust evidence for the superiority of this approach currently. Nevertheless, our review highlights the promise of CPUS for evaluating patients suspected of having PE.

The strengths of our systematic review and meta-analysis include its comprehensive scope and search and the quality of the studies was generally high. All studies ensured proper blinding of the sonographer to the diagnostic test results. Limitations include the moderate heterogeneity between the included studies which puts some caution to the interpretation of these results. These data reflect the methodological diversity of the analyzed studies, as described previously (study setting, design, sampling, blinding, sonographer’s expertise, and reference standard). There are no universal standardized approaches to US delivery and reporting and this lack of core outcomes sets (COS) contributes to the heterogeneity between trials [30]. We also recognize that variability was inherent in this meta-analysis because bedside lung or cardiac ultrasound findings are not pathognomonic for PE. Lastly, clinically derived PE diagnosis is a subjective reference standard [31]. Subjectivity in the studies we included was minimized by using a predefined protocol for most studies [17, 18, 22, 23] based on internationally accepted diagnostic criteria. Since patients are likely to have more than one abnormal finding per ultrasound, we used the individual patient as the unit of analysis (abnormal finding versus none) and not the individual ultrasound findings. Clinically, it is also useful to know the accuracy of individual ultrasound findings as it is plausible that some findings are better indicators of PE than others.

## Conclusions

Our findings suggest that cardiopulmonary ultrasonography has potential to be used in PE diagnostics when CTPA is unsuitable or unavailable. CPUS may be highly relevant in low resource settings as it is cheaper and is less human resource dependent compared to CTPA. Based on the current evidence, a global harmonization of US diagnostic criteria for PE is required, potentially within worldwide consensus guidelines that will allow specifications of essential practice standards for low resource settings. Given the complexity of clinical investigation, future studies should investigate the impact of a point of care CPUS on diagnostic certainty, wider resource and test use, and the effects on patient pathways and treatment plans.

## Supporting information

S1 Appendix Link to protocol. https://www.crd.york.ac.uk/PROSPERO. ID = CRD42018099925.
(TXT)

S1 Fig Pooled receiver operator characteristic curve of CPUS in diagnosing PE. Summary receiver operating characteristic (SROC) curve illustrating the pooled sensitivity and specificity of the two reference standards used (CTPA [black] and Experts clinicians audit [red]).
(DOCX)

S2 Fig Proposed algorithm for pulmonary embolism diagnosis. The proposed algorithm that considers the use of the triple point of care ultrasonography of the heart, lung and leg veins for the diagnosis of PE.
(DOCX)

S1 File PRISMA checklist. (DOC)

S2 File Search strategies and search terms. (DOCX)

## References

[1] Jha AK, Larizgoitia I, Audera-Lopez C, Prasopa-Plaisier N, Waters H BD. The global burden of unsafe medical care: analytic modeling of observational studies. BMJ Qual Saf. 2013;22:809–15. 10.1136/bmjqs-2012-001748 24048616

[2] Goldhaber SZ, Visani L, De Rosa M. Acute pulmonary embolism: clinical outcomes in the International Cooperative Pulmonary Embolism Registry (ICOPER). Lancet (London, England) [Internet]. 1999 4 24;353(9162):1386–9. Available from: http://search.ebscohost.com/login.aspx?direct=true&db=mdc&AN=10227218&site=ehost-live&authtype=ip,shib&user=s1523151 10.1016/s0140-6736(98)07534-5 10227218

[3] Riedel M. VENOUS THROMBOEMBOLIC DISEASE: Acute pulmonary embolism 1: pathophysiology, clinical presentation, and diagnosis. Heart [Internet]. 2001;85(2):229–40. Available from: http://heart.bmj.com/cgi/doi/10.1136/heart.85.2.229 11156681 10.1136/heart.85.2.229 PMC1729607

[4] Squizzato A, Galli L, Gerdes VEA. Point-of-care ultrasound in the diagnosis of pulmonary embolism. Crit Ultrasound J [Internet]. 2015 5 27;7:7 Available from: http://search.ebscohost.com/login.aspx?direct=true&db=mdc&AN=26034556&site=ehost-live&authtype=ip,shib&user=s1523151 10.1186/s13089-015-0025-5 26034556 PMC4447771

[5] A. S, L. G. Point-of-care ultrasound in the diagnosis of pulmonary embolism. Crit Ultrasound J [Internet]. 2015;7(1):7. Available from: http://www.springer.com/medicine/critical+care+and+emergency+medicine/journal/13089?cm_mmc=AD-_-CFP-_-ITA12667_V1-_-13089 10.1186/s13089-015-0025-5 PMC4447771 26034556

[6] Squizzato A, Rancan E, Dentali F, Bonzini M, Guasti L, Steidl L, et al Diagnostic accuracy of lung ultrasound for pulmonary embolism: a systematic review and meta-analysis. J Thromb Haemost JTH [Internet]. 2013 7;11(7):1269–78. Available from: http://search.ebscohost.com/login.aspx?direct=true&db=mdc&AN=23574640&site=ehost-live&authtype=ip,shib&user=s1523151 10.1111/jth.12232 23574640

[7] Adhikari S, Amini R, Stolz L BM. Impact of point-of-care ultrasound on quality of care in clinical practice. Reports Med Imaging. 2014;7:81–93.

[8] Mercaldi CJ LS. Ultrasound guidance decreases complications and improves the cost of care among patients undergoing thoracentesis and paracentesis. Chest. 2013;143(2):532–8. 10.1378/chest.12-0447 23381318

[9] Smallwood N, Dachsel M. Point-of-care ultrasound (POCUS): Unnecessary gadgetry or evidence-based medicine? Clin Med J R Coll Physicians London. 2018;18(3):219–24. 10.7861/clinmedicine.18-3-219 PMC6334078 29858431

[10] Moher D, Liberati A, Tetzlaff J AD. The PRISMA Group (2009). Preferred Reporting Items for Systematic Reviews and Meta-Analyses: The PRISMA Statement. Ann Intern Med. 2009;151(4):264–9. 10.7326/0003-4819-151-4-200908180-00135 19622511

[11] Whiting PF, Rutjes AW, Westwood ME, Mallett S, Deeks JJ RJ, Leeflang MM, et al Bossuyt PM Q-2 G. QUADAS-2: a revised tool for the quality assessment of diagnostic accuracy studies. Ann Intern Med. 2011;155(8):529–536. 10.7326/0003-4819-155-8-201110180-00009 22007046

[12] The Nordic Cochrane Centre TCC. Review Manager 5 (RevMan 5). 2014. p. version 5.3.

[13] R Core Team. R: a language and environment for statistical computing. 2017.

[14] Zamora J, Abraira V, Muriel A, Khan K CA. Meta-DiSc: a software for meta-analysis of test accuracy data. BMC Med Res Methodol. 2006;6:31 10.1186/1471-2288-6-31 16836745 PMC1552081

[15] Deeks JJ, Macaskill P IL. The performance of tests of publication bias and other sample size effects in systematic reviews of diagnostic test accuracy was assessed. J Clin Epidemiol. 2005;58:882–93. 10.1016/j.jclinepi.2005.01.016 16085191

[16] Egger M, Smith GD, Schneider M MC. Bias in meta-analysis detected by a simple, graphical test. Br Med J. 1997;315:629–34. 9310563 10.1136/bmj.315.7109.629 PMC2127453

[17] Laursen CB, Sloth E, Lambrechtsen J, Lassen AT, Madsen PH, Henriksen DP, et al Focused sonography of the heart, lungs, and deep veins identifies missed life-threatening conditions in admitted patients with acute respiratory symptoms. Chest. 2013;144(6):1868–75. 10.1378/chest.13-0882 23948720

[18] Laursen CB, Sloth E, Lassen AT, Christensen R, Lambrechtsen J, Madsen PH. Point-of-care ultrasonography in patients admitted with respiratory symptoms: a single-blind, randomised controlled trial. 2014;2(8). 10.1016/S2213-2600(14)70135-3 24998674

[19] Nazerian P, Vanni S, Volpicelli G, Gigli C, Zanobetti M, Bartolucci M, et al Accuracy of point-of-care multiorgan ultrasonography for the diagnosis of pulmonary embolism. Chest [Internet]. 2014 5;145(5):950–7. Available from: http://search.ebscohost.com/login.aspx?direct=true&db=mdc&AN=24092475&site=ehost-live&authtype=ip,shib&user=s1523151 10.1378/chest.13-1087 24092475

[20] Koenig S, Chandra S, Alaverdian A, Dibello C, Mayo PH, Narasimhan M. Ultrasound assessment of pulmonary embolism in patients receiving CT pulmonary angiography. Chest. 2014;145(4):818–23. 10.1378/chest.13-0797 24178672

[21] Bataille B, Riu B, Ferre F, Moussot PE, Mari A, Brunel E, et al Integrated use of bedside lung ultrasound and echocardiography in acute respiratory failure: a prospective observational study in ICU. Chest [Internet]. 2014 12;146(6):1586–93. Available from: http://search.ebscohost.com/login.aspx?direct=true&db=mdc&AN=25144893&site=ehost-live&authtype=ip,shib&user=s1523151 10.1378/chest.14-0681 25144893

[22] Zanobetti M, Scorpiniti M, Gigli C, Nazerian P, Vanni S, Innocenti F, et al Point-of-Care Ultrasonography for Evaluation of Acute Dyspnea in the ED. Chest [Internet]. 2017 6;151(6):1295–301. Available from: http://search.ebscohost.com/login.aspx?direct=true&db=mdc&AN=28212836&site=ehost-live&authtype=ip,shib&user=s1523151 10.1016/j.chest.2017.02.003 28212836

[23] Ahn JH, Jeon J, Toh HC, Noble VE, Kim JS, Kim YS, et al SEARCH 8Es: A novel point of care ultrasound protocol for patients with chest pain, dyspnea or symptomatic hypotension in the emergency department. PLoS One. 2017;12(3):1–13. 10.1371/journal.pone.0174581 PMC5371336 28355246

[24] Baujat Bertrand, Cédric Mahé Jean‐Pierre Pignon CH. A graphical method for exploring heterogeneity in meta‐analyses: application to a meta‐analysis of 65 trials. Stat Med. 2002;21(18):2641–52. 10.1002/sim.1221 12228882

[25] Fields JM et al Transthoracic Echocardiography for Diagnosing Pulmonary Embolism: A Systematic Review and Meta-Analysis. J Am Soc Echocardiogr. 2017;30(7):714–723.e4. 10.1016/j.echo.2017.03.004 28495379

[26] A. S, E. R, F. D. Diagnostic accuracy of lung ultrasound for pulmonary embolism: A meta analysis. Blood Transfus [Internet]. 2012;10(SUPPL. 4):s118 Available from: http://www.bloodtransfusion.it

[27] Lichtenstein DA, Mezière GA. Relevance of lung ultrasound in the diagnosis of acute respiratory failure: the BLUE protocol. Chest [Internet]. 2008 7;134(1):117–25. Available from: http://search.ebscohost.com/login.aspx?direct=true&db=mdc&AN=18403664&site=ehost-live&authtype=ip,shib&user=s1523151 10.1378/chest.07-2800 18403664 PMC3734893

[28] Frank L, Quint LE. Chest CT incidentalomas: Thyroid lesions, enlarged mediastinal lymph nodes, and lung nodules. Cancer Imaging. 2012;12(1):41–8. 22391408 10.1102/1470-7330.2012.0006 PMC3335330

[29] Konstantinides S V., Meyer G, Bueno H, Galié N, Gibbs JSR, Ageno W, et al 2019 ESC Guidelines for the diagnosis and management of acute pulmonary embolism developed in collaboration with the European respiratory society (ERS). Eur Heart J. 2020;41(4):543–603. 10.1093/eurheartj/ehz405 31504429

[30] Kirkham JJ, Gorst S, Altman DG, Blazeby JM, Clarke M, Devane D et al Core Outcome Set–STAndards for Reporting: The COS-STAR Statement. PLoS Med. 2016;13(10):e1002148 10.1371/journal.pmed.1002148 27755541 PMC5068732

[31] Bertens LCM, Broekhuizen BDL, Naaktgeboren CA, Rutten FH, Hoes AW et al Use of Expert Panels to Define the Reference Standard in Diagnostic Research: A Systematic Review of Published Methods and Reporting. PLOS Med. 2013;10(10):e1001531 10.1371/journal.pmed.1001531 24143138 PMC3797139

