# Supplementary figures and images for: Diagnostic accuracy of combined thoracic and cardiac sonography for the diagnosis of pulmonary embolism: A systematic review and meta-analysis
Source: PLoS One. 2020 Sep 28;15(9):e0235940. doi: 10.1371/journal.pone.0235940 (PMC7521742; doi:10.1371/journal.pone.0235940)

**
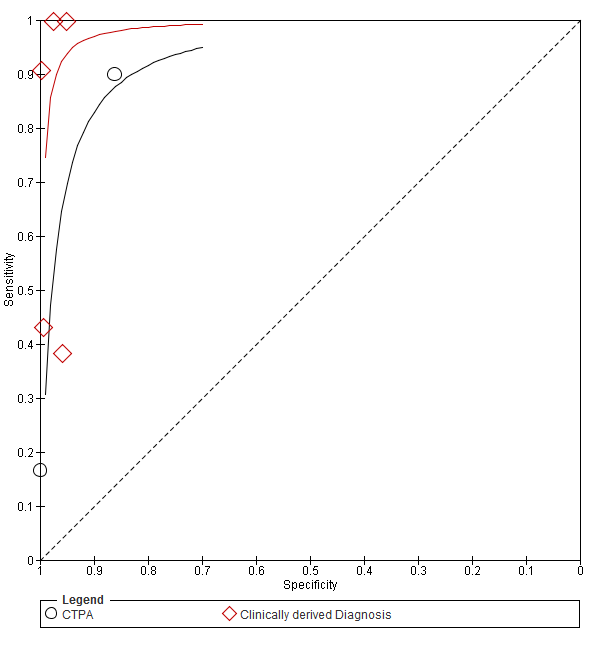
**

Supplement: S1 Fig — Summary receiver operating characteristic (SROC) curve illustrating the pooled sensitivity and specificity of the two reference standards used (CTPA [black] and Experts clinicians audit [red]). (DOCX) [file pone.0235940.s002.docx]

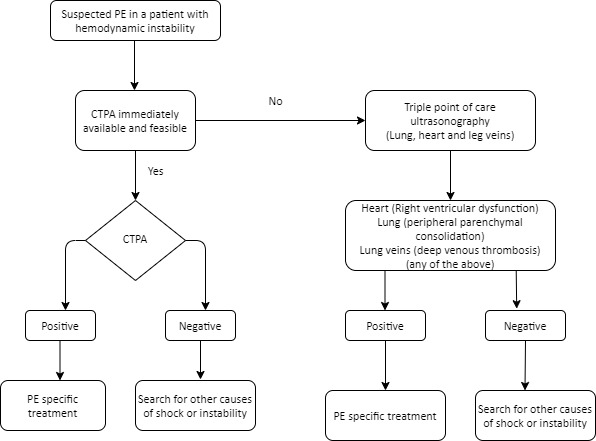

Supplement: S2 Fig — The proposed algorithm that considers the use of the triple point of care ultrasonography of the heart, lung and leg veins for the diagnosis of PE. (DOCX) [file pone.0235940.s003.docx]
