# Supplementary material for: Diagnostic accuracy of combined thoracic and cardiac sonography for the diagnosis of pulmonary embolism: A systematic review and meta-analysis
Source: PLoS One. 2020 Sep 28;15(9):e0235940. doi: 10.1371/journal.pone.0235940 (PMC7521742; doi:10.1371/journal.pone.0235940)
Supplement: S2 File — (DOCX) [file pone.0235940.s005.docx]

| Terms referring to the clinical feature of interest |  | Terms synonymous with or likely to include ultrasound | Terms referring to pulmonary embolism |
| --- | --- | --- | --- |
| breathless*  OR  dyspn*  OR  Tachypn*  OR  “Difficulty in breathing”  OR  “Shortness of breath”  OR  “Respiratory distress”  OR  “Air hunger”  OR  “Respiratory failure” | And | sonogr*  OR  ultraso*  OR  “Focused ultraso*”  OR  “Bedside ultraso*”  OR  “Point of care ultraso*”  OR  “Emergency ultraso*”  OR  “Abbreviated ultraso*”  OR  “portable ultraso*”  OR  “hand-held ultraso*” | “pulmonary embol*”  OR  “pulmonary thrombo embol*” |

## **MEDLINE search strategy (Initial search June 6^th^, 2017; updated search February 28^th^ 2020)**

S1

breathless* OR dyspn* OR Tachypn* OR “Difficulty in breathing” OR “Shortness of breath” OR “Respiratory distress” OR “Air hunger” OR “Respiratory failure”

S2

“Point of care ultraso*” OR sonogr* OR “Focused ultraso*” OR “Bedside ultraso* OR ultraso* OR “Emergency ultraso*” OR “Abbreviated ultraso*” OR “portable ultraso*” OR “hand-held ultraso*”

S3

S1 AND S2

S4

“pulmonary thrombo embol*” OR “pulmonary embol*”

S5

S4 AND S3
