# Supplementary material for: Diagnostic accuracy of combined thoracic and cardiac sonography for the diagnosis of pulmonary embolism: A systematic review and meta-analysis
Source: PLoS One. 2020 Sep 28;15(9):e0235940. doi: 10.1371/journal.pone.0235940 (PMC7521742; doi:10.1371/journal.pone.0235940)
Supplement: Attachment — Submitted filename: Response to Reviewers.docx [file pone.0235940.s006.docx]

Jacqueline Wanjiku Kagima
Liverpool School of Tropical Medicine
13372, Nairobi

26/06/2020

Muhammad Adrish
Academic Editor
PLOS ONE

Dear Muhammad Adrish,

**RE:	Response to reviewers**

Thank you for the opportunity to revise our manuscript “PONE-D-20-19213- *Diagnostic accuracy of combined thoracic and cardiac sonography for the diagnosis of pulmonary embolism: a systematic review and meta-analysis*”. We appreciate the careful review and constructive suggestions. It’s our belief that the manuscript is substantially improved after making the suggested edits.

Following this letter are the editor and reviewer comments raised through your email dated 14^th^ July 2020 regarding the manuscript with our point by point responses. Changes made in the manuscript are marked using track changes.

We hope that the revised version is now suitable for publication and look forward to hearing from you in due course.

Yours sincerely

Dr Jacqueline Wanjiku Kagima

Liverpool School of Tropical Medicine

[Jacikagima@gmail.com](mailto:Jacikagima@gmail.com)

[Jacqueline.Kagima@lstmed.ac.uk](mailto:Jacqueline.Kagima@lstmed.ac.uk)

**RESPONSE TO REVIEWER 1;**

**Response to Reviewer 1: An interesting manuscript in its field, I suggest that the authors add a figure with their proposed algorithm when all tools for the diagnosis of pulmonary embolism are not available.**

Thank you for your review of our paper and comment. We agree and have added a proposed algorithm in our discussion (**page 16, line 264-265**) and added the figure under supporting information (**S2 Fig**).

In this paper, we report that that cardiopulmonary ultrasonography has potential to be used in pulmonary embolism (PE) diagnostics when computed tomography pulmonary angiography (CTPA) is unsuitable or unavailable. This is significant because cardiopulmonary ultrasound may be highly relevant in low resource settings as it is cheaper and is less human resource dependent compared to CTPA. Taking this into consideration, we propose an algorithm that considers the use of the triple point of care ultrasonography of the heart, lung and leg veins in the diagnosis of PE as shown in the Figure below.

Proposed algorithm that considers the use of the triple point of care ultrasonography of the heart, lung and leg veins for the diagnosis of PE

**RESPONSE TO REVIEWER 2;**

**The authors conducted a meta-analysis of an important clinical problem -i.e., to look at validity of ultrasound to diagnose pulmonary embolism using two different reference criteria; the stronger one - CT chest angiography - being used in only two studies, unfortunately; the outcome is very helpful to address this difficult diagnosis in low resource settings where CT is perhaps not available, while ultrasound of heart, lungs and legs combined appear very helpful to address this diagnostic dilemma. The study team used all standard ways to do data extraction, perform quality assessment of included studies, looking at forest plots, funnel plots to explore possible publication bias, using standard reporting and having registered the study at PROSPERO, all meeting the criteria for high quality**

Thank you for your review of our paper. We appreciate these comments. We agree indeed that ultrasound of the heart, lungs and legs combined may be very helpful in in low resource settings especially when CTPA is not immediately available or feasible.

**EDITOR COMMENTS**

We have addressed the editorial points as follows;

**1. Please ensure that your manuscript meets PLOS ONE's style requirements, including those for file naming.**

Thank you. We have corrected these with track changes on the manuscript.

**2. At this time, we ask that you please provide the full search strategy and search terms for at least one database used as Supplementary Information.**

This information is provided under supporting information, S2 File. Search strategies and search terms, page 22.

**3. In the Methods section, please provide the specific information extracted from the articles selected in your study in the 'Study selection and data extraction sub-section.**

Data extracted information has been provided in study selection and data extraction section (page 5, line 91 to 94). “Data was extracted on the study setting, sampling methods, characteristics of the study design, reference standard used in the diagnosis of PE and additional tests done, type of ultrasound machine used and the organs scanned and sonographers qualifications, experience and blinding”.
